# Hybrid RPA:DFT Approach for Adsorption on Transition Metal Surfaces: Methane and Ethane on Platinum (111)

**DOI:** 10.1021/acs.jctc.3c01308

**Published:** 2024-02-08

**Authors:** Christopher Sheldon, Joachim Paier, Denis Usvyat, Joachim Sauer

**Affiliations:** † Institut für Chemie, Humboldt-Universität zu Berlin, Unter den Linden 6, Berlin 10099, Germany; ‡ Fritz-Haber-Institut der Max-Planck-Gesellschaft, Faradayweg 4, Berlin 14195, Germany; § Lehrstuhl für Theoretische Chemie, Friedrich-Alexander-Universität Erlangen-Nürnberg, Egerlandstrasse 3, Erlangen 91058, Germany

## Abstract

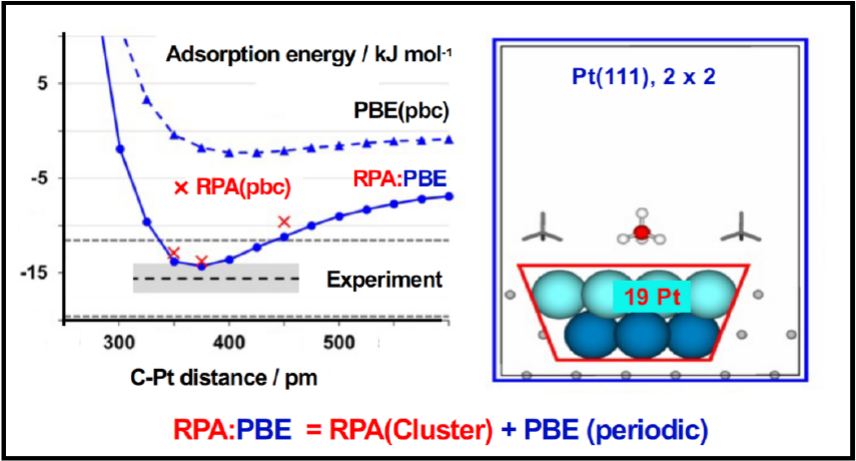

The hybrid QM:QM approach is extended to adsorption on transition metal surfaces. The random phase approximation (RPA) as the high-level method is applied to cluster models and, using the subtractive scheme, embedded in periodic models which are treated with density functional theory (DFT) that is the low-level method. The PBE functional, both without dispersion and augmented with the many-body dispersion (MBD), is employed. Adsorption of methane and ethane on the Pt(111) surface is studied. For methane in a 2 × 2 surface cell, the hybrid RPA:PBE and RPA:PBE+MBD results, −14.3 and −16.0 kJ mol^–1^, respectively, are in close agreement with the periodic RPA value of −13.8 kJ mol^–1^ at significantly reduced computational cost (factor of ∼50). For methane and ethane, the RPA:PBE results (−14.3 and −17.8 kJ mol^–1^, respectively) indicate underbinding relative to energies derived from experimental desorption barriers for relevant loadings (−15.6 ± 1.6 and −27.2 ± 2.9 kJ mol^–1^, respectively), whereas the hybrid RPA:PBE+MBD results (−16.0 and −24.9 kJ mol^–1^, respectively) agree with the experiment well within experimental uncertainty limits (deviation of −0.4 ± 1.5 and +2.3 ± 2.9 kJ mol^–1^, respectively). Finding a cluster that adequately and robustly represents the adsorbate at the bulk surface is important for the success of the RPA-based QM:QM scheme for metals.

## 1 Introduction

Accurate modeling of chemical reactions from first-principles is one of the major goals of quantum chemistry, but for elementary adsorption and reaction steps on (transition) metal surfaces this remains a significant challenge. Density functional theory (DFT), the workhorse of computational catalysis and material science, does not provide “chemical accuracy” (±4 kJ mol^–1^),^1^ which is required to make reactivity predictions that are useful for modeling heterogeneous catalysis.^2^

Mean absolute errors (MAE) of about 25 kJ mol^–1^ have been reported for reactions on metal surfaces,^3^ whereas maximum errors (ME) can be as large as 55 kJ mol^–1^. Even advanced variants like the Bayesian error estimation functional with van der Waals correlation contributions (BEEF–vdW) show MAE of about 30 kJ mol^–1^ for reactions on transition metal surfaces,^3,4^ with MEs of 115–130 kJ mol^–1^.^3,4^

Wave function methods generally provide a better description (and can be systematically improved in principle) but at a much increased computational cost. For periodic systems, these can be prohibitive if additional approximations are not applied. The hybrid QM:QM approach^5,6^ offers a solution to these problems. The computationally efficient low-level method, DFT, is applied to the entire system, while a high-level correction, where wave function methods are used, is applied to a subset of this, in the form of a cluster. The hybrid QM:QM approach has been successfully applied to many nonconducting periodic systems,^2,7−9^ but the difficulty comes as soon as transition metal atoms with different spin states in a narrow energy range are involved, necessitating costly multireference methods. The hybrid QM:QM treatment is facing its limits for conducting systems, where the choice of the high-level method, or cluster to which it is applied, or both, is not straightforward.^10−14^

This preclusion of metals is unfortunate, as many important chemical reactions occur on metal surfaces. One exemplar reaction is the dehydrogenation step of the reformation process, which occurs on supported Pt catalysts. As a model of reduced complexity,^15^ the Pt(111) surface is studied both experimentally and computationally.^16,17^ As a first step, the alkane must adsorb, chiefly through the dispersion interaction, which is unaccounted for by standard DFT. One way to include this is to apply additive, post-SCF dispersion corrections (+D),^3,18^ or to amend the density functional to include them (i.e., vdW-functionals).^19,20^ These offer a reasonable description but, in combination with generalized gradient approximation (GGA)-type functionals, fail to reach the accuracies required for application to reaction kinetics, which exponentially magnifies the errors. Generally, chemical accuracy is required for such problems, well beyond the current capability of DFT including dispersion. This calls for implementation of a hybrid QM:QM scheme for (transition) metals. That this is possible, albeit not straightforward, has been demonstrated by Pettersson and coworkers who used DFT with a hybrid functional (M06) as the high-level method.^21^ They have applied a hybrid M06:PBE+D3 scheme with PBE+D3 as the low-level method to adsorption on transition metal surfaces.^21^

Whereas hybrid functionals provide an improvement compared to GGA-type functionals, there is an increasing number of studies that show the need for climbing the Jacob’s ladder to the random phase approximation (RPA)^22−26^ to reach, or get close to, chemical accuracy.^27−30^ RPA for metals poses several issues. Metals have, by definition, a zero-width band gap, with many close-lying energy levels. For any “finite-order” perturbative method, the correlation energy diverges for zero-width energy gaps.^31,32^ Some of us have previously applied RPA to the study of CH_4_ on the Pt(111) surface and obtained chemically accurate adsorption energies for two, physically relevant, coverages.^29^ However, the scaling of the computational cost,^22,33^ O(N^4^) with respect to the number of plane waves and O(N^2^) with respect to *k*-points, prevented us from studying larger systems. Other implementations have similarly high costs.^34^

Here, we present a hybrid RPA:DFT scheme to reduce the immense computational costs of a full RPA calculation with periodic boundary conditions. We study the adsorption of CH_4_ and C_2_H_6_ on Pt(111), for which reliable experimental data are available.^35^ We calculate hybrid RPA:DFT potential energy curves, which are corrected for the basis set superposition error (BSSE). For CH_4_/Pt(111), comparison of our RPA:DFT results with previously obtained periodic RPA results allows us to assess the accuracy of our approach.^29^

A hybrid RPA:DFT scheme has been applied before to approximate single-point RPA energies for CO/Cu(111).^36^ Very recently, Carter and coworkers have used electronic embedding of clusters to study H_2_ dissociation on the Cu(111) surface and were able to reduce the computational cost by 2 orders of magnitude.^37^

## 2 Methods

### 2.1 Hybrid QM:QM Calculations with Counterpoise Correction

Our hybrid QM:QM approach uses the subtractive scheme^38−40^ with periodic boundary conditions (pbc).^39^ The hybrid energy E_HL:LL_(pbc) is
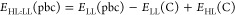
1where *E*_LL_(pbc) is the total energy per cell of the periodic system calculated using the low-level method, and *E*_LL_(C) and *E*_HL_(C) are the low- and high-level energies of the finite cluster, respectively. The hybrid energy may be conceived as a high-level correction ΔHL(C) to the energy of the periodic system using a low-level method:
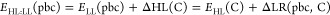
2
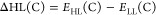
3

Alternatively, but entirely equivalently, the hybrid energy may be conceived as adding a low-level, long-range correction ΔLR(pbc, C) to the high-level energy of the cluster:
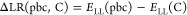
4

Since we employ an atomic orbital basis set for cluster calculations, there is a need to correct for the BSSE.^41^ The interaction energy between two monomers A and B is defined as
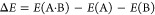
5

We correct for the BSSE by using the counterpoise correction (CPC) scheme:^41^
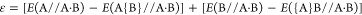
6where “//” denotes “at the structure of”, meaning that the energies are computed at the structure of the A·B complex. The *E*(A{B}//A·B) and *E*({A}B//A·B) refer to the A and B entities, respectively, in the full basis of the A·B complex. The BSSE correction is then obtained by
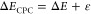
7

The pbc calculations in our approach are carried out using a plane wave basis set, eliminating the need for BSSE corrections.

### 2.2 Hybrid QM:QM Adsorption Energies

The adsorption energy per molecule for an adsorbate layer of *N* molecules per unit cell is defined as
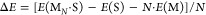
8where M*_N_*·S is the adsorbate–surface system, S is the bare surface, and M is the molecule in the gas phase, each at their equilibrium structure. The adsorption energy may be divided into the adsorbate–surface interaction ΔE* and the lateral interactions Δ*E*_lat_:
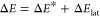
9according to
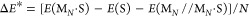
10and
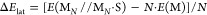
11where *E*(M*_N_*//M*_N_*·S) is the energy of the adsorbate layer at the structure of the adsorbate–surface system M*_N_*·S. With decreasing coverage, the lateral interaction tends toward zero and the adsorption energy is exclusively defined by the adsorbate–surface interaction.

For the energies in eqs 8–11, the hybrid QM:QM values are computed by applying the QM:QM subtractive scheme of eq 1 to each of these equations:
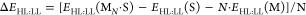
12

## 3 Experiments and Models

### 3.1 Experiments

We will compare our computed results with the temperature-programmed desorption experiments of Tait et al.^35^ The reported desorption energies are Arrhenius activation energies, *E*_A_. Following previous work (see also Sheldon et al.^29^ ), we first convert them into heats of adsorption, Δ*H*_ads_(T), at temperature *T* (*R* is the gas constant),
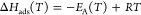
13and then, taking into account the zero-point vibrational energy, Δ*E*_ZPV_, and the thermal vibrational contributions to the energy, Δ*E*_therm_, into “experimentally derived” reference energies, see Table 1:
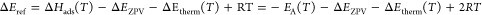
14

**Table 1:** Adsorption Energies Derived from Observed Desorption Barriers for Θ = ^1^/_4_. All Energies in kJ mol^–1^

	– *E*_A_ obsd^a^	*T*/K	2RT	Δ*E*_ZPV_ calc.^b^	Δ*E*_therm_ calc.^b^	Δ*E*_ref_ obsd.
CH_4_	–15.5 ± 1.5	63	1.04	–0.77	–0.35	–15.6 ± 1.5
C_2_H_6_	–29.4 ± 2.9	106	1.76	–1.17	0.68	–27.2 ± 2.9

a Ref (35), assuming that 1/4 ML coverage is saturated coverage; 10% uncertainty of the inversion analysis.^42^

b PBE+MBD

We calculated the vibrational energies needed in the harmonic approximation using PBE+MBD wavenumbers.

### 3.2 Periodic Models

**Figure 1:**
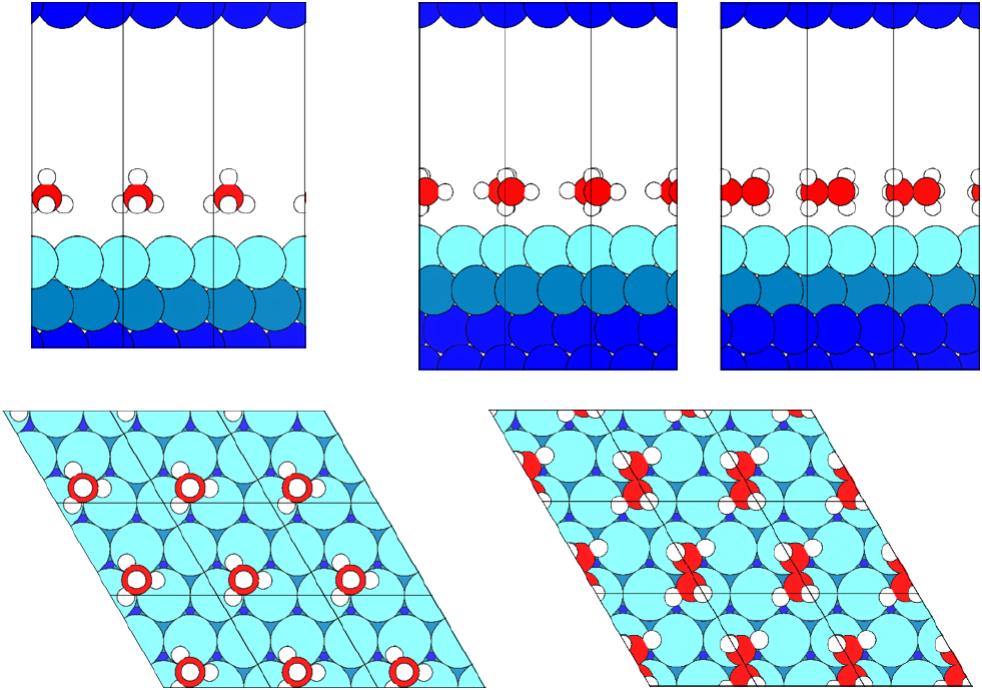
Pt(111) surface (2 × 2) cells) with ^1^/_4_ ML adsorbate coverage viewed in parallel (top) and perpendicular (bottom) to the surface. Left: CH_4_, right: C_2_H_6_ (two side views, rotated by 90°). Color code: platinum–blue (light – first-layer cluster atoms, turquoise – second-layer cluster atoms, dark – third- and fourth-layer atoms), carbon–red, oxygen–orange, and hydrogen–white.^43^.

For CH_4_/Pt(111), we adopt the same 3-layered (2 × 2) Pt(111) cell that we had previously used for studying RPA at ^1^/_4_ ML coverage^29^ (see Figure 1, left). One monolayer (ML) is (formally) defined as one adsorbed molecule per surface Pt atom.^29^ For C_2_H_6_/Pt(111), also presented in Figure 1, a 4-layer slab model is used.

### 3.3 Clusters

**Figure 2:**
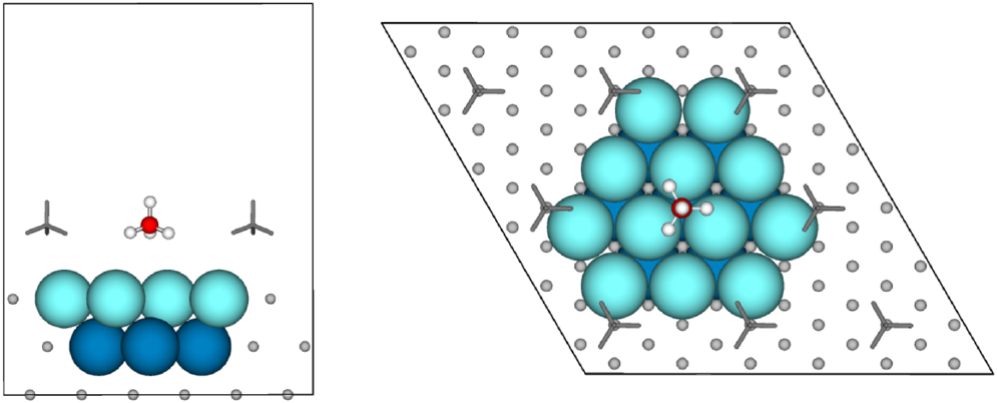
CH_4_/Pt_19_(12,7) cluster embedded in the Pt(111) surface at ^1^/_4_ ML coverage viewed from the side (left) and from top (right). Color code: platinum – light blue (first layer) and turquoise (second layer); carbon – red; hydrogen – white.^44^

Clusters were cut from a PBE+MBD-optimized (2 × 2) Pt(111) slab. They are named Pt_*n*_(*a*,*b*,...), where *n* is the total number of Pt atoms in the cluster, *a* is the number in the top layer of the cluster, *b* is the number in the second layer, and so on. Figure 2 shows the Pt_19_(12,7) cluster embedded in the Pt(111) surface, for the clusters studied (see Section S1). The same geometric structures were used for calculations both with and without periodic boundary conditions (pbc). With pbc, the clusters were placed in 20^3^ Å^3^ cubic cells, which were found to be sufficiently large to avoid image interaction (see Table S2.1). Likewise, isolated, gaseous alkane molecules were modeled using identical cells (20^3^ Å^3^). All pbc calculations use a 14 Å vacuum height (i.e., the distance between the slab surface and its repeated image).

## 4 Computational Details

### 4.1 VASP Calculations

#### 4.1.1 DFT

Plane wave DFT calculations were performed using the projector-augmented wave (PAW) method,^45,46^ as implemented in the Vienna ab initio simulation package (VASP).^47^ The PAW pseudopotential used to describe the electron–ion interaction for Pt includes the 4f electrons resulting in 10 valence electrons: [Xe,4f^14^]5d^9^6s^1^. Two partial waves were used for each orbital and their cutoff radius was 2.5 au for both the 5d and 6s states. For C, 4 valence electrons ([He]2s^2^2p^2^) were considered. The partial wave cutoff radii were 1.2 and 1.5 au for 2s and 2p, respectively. For O, 6 valence electrons ([He]2s^2^2p^4^) were considered. The partial wave cutoff radii were 1.2 and 1.52 au for 2s and 2p, respectively. For the 1s orbital of H, a partial wave cutoff radius of 1.1 au was used. These pseudopotentials were used for all structure optimizations.

An electronic energy threshold of 1 × 10^–6^ eV, a 6 × 6 × 1 *k*-point mesh, and a plane wave energy cutoff, *E*_cutoff_, of 400 eV were applied. Calculations involving Pt used first-order Methfessel–Paxton smearing with a smearing width of 0.2 eV, while those on isolated alkanes used Gaussian smearing with a smearing width of 0.05 eV. For the isolated alkanes and clusters, only the Γ-point was sampled. To enable more direct comparison with unrestricted, Gaussian basis set calculations, spin-polarized calculations were performed, unless otherwise stated. The difference with non-spin-polarized calculations is generally less than 0.1 kJ mol^–1^ and at most 0.5 kJ mol^–1^ for Pt_46_(27,19) (cf. Table S2.2).

Structure optimizations were performed until all forces on relaxed atoms were converged to below 0.01 eV Å^–1^ (0.194 mE_h_ Bohr^–1^). The bottom two Pt layers were frozen in position to mimic the bulk. The conjugate gradient method was used with cell shape and volume kept constant.

The PBE^48,49^ density functional was used throughout with dispersion corrections, denoted PBE+D. Grimme’s D2^50^ and D3,^18^ Tkatchenko–Scheffler’s many-body dispersion (MBD),^51−53^ and Steinmann–Corminbouef’s (dDsC)^54,55^ dispersion corrections were used.

#### 4.1.2 RPA

The periodic RPA calculations (CH_4_/Pt(111)) were performed according to Sheldon et al.^29^ using the projector-augmented wave (PAW) method,^45,46^ as implemented in the Vienna ab initio simulation package (VASP).^47^ All calculations used an electronic energy threshold of 1 × 10^–8^ eV and a *E*_cutoff_ of 500 eV. Calculations involving Pt used first-order Methfessel–Paxton smearing with a smearing width of 0.2 eV, while those on isolated alkanes used Gaussian smearing with a smearing width of 0.05 eV.

For RPA, *GW* PAW pseudopotentials^56^ were used with identical core and valence definitions as the above but improved scattering properties for unoccupied states (PBE cores, as in VASP 5.4). For Pt, the partial wave cutoff radii were 2.4 au for both the 5d and 6s states. For C, the partial wave cutoff radii were 1.2 and 1.5 au for 2s and 2p, respectively. For the 1s orbital of H, a cutoff radius of 0.95 au was used. A frequency integration grid density containing 18 and 12 points were used for Pt and isolated CH_4_ calculations, respectively. For further details of RPA, see Sheldon et al.^29^

### 4.2 TURBOMOLE Calculations for Clusters

#### 4.2.1 DFT

The calculations were performed using the resolution of identity (RI)-DFT module^57,58^ available in 7.3.1 version of the TURBOMOLE program.^59^ Restricted DFT was used for singlet calculations, high-spin calculations used unrestricted DFT. Additionally, effective core potentials (def2-ECPs) were used for the 60 core electrons of platinum.^60^ For C, H, and Pt atoms, def2-QZVPP basis sets were used, with the corresponding auxiliary bases.^60−62^ Energies were converged to within 10^–7^ Ha. An automatic orbital shift of 0.4 eV and heavy damping was applied to aid SCF convergence.

#### 4.2.2 RPA

Using orbitals from the aforementioned DFT calculations, the RPA calculations employed the resolution of identity (RI)-RPA module.^63,64^ The “frozen-core” approximation was applied, with orbitals below 2 Ha considered to be core. (RI)-MP2 and (RI)-CC def2-QZVPP auxiliary, correlation basis sets^60^ were used, as recommended in the literature.^65,66^ Counterpoise corrections (CPC) were performed on all cluster calculations.^41^ The number of integration points necessary for calculating the RPA correlation energy depended on the cluster size; 100 and 220 integration points were required to achieve 0.1 kJ mol^–1^ convergence in adsorption energy for Pt_19_ and Pt_28_, respectively. For details of the numbers of occupied and virtual orbitals, and comparison to plane wave results, see Table S4.6. Additionally, the DFT orbitals were used to obtain their corresponding Hartree–Fock energy, performing a single elementary step, as implemented in the dscf module.^67^

## 5 Results and Discussion

### 5.1 DFT Results for Clusters Compared to Periodic Models

For CH_4_ on Pt(111), the periodic PBE+D results, pbc_lat_ (see Table S2.3) approach the experimentally derived adsorption energy at ^1^/_4_ ML coverage of −15.6 kJ mol^–1^ (see Table 1)^29,35^ in the series (kJ mol^–1^) D = D2 (−35.6), D3 (−24.9), dDsC (−18.9), and MBD (−14.7). It is well-known that D2 overbinds for metals.^68^ Although D3 is a substantial improvement, only dDsC and in particular MBD get close to the experimentally derived adsorption energy.^69^

**Figure 3:**
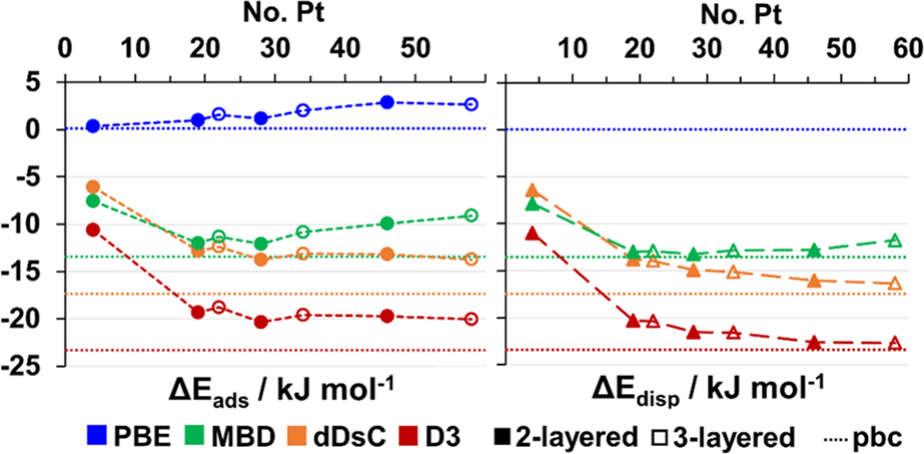
Adsorption energy for CH_4_ on Pt_*n*_ clusters as a function of cluster size. Left: circles show the total adsorption energy Δ*E*_ads_. Right: triangles show the dispersion component Δ*E*_disp_. Full and hollow markers are for 2- and 3-layered clusters, respectively. The pbc values are shown as straight, dashed lines (lateral interactions removed, cf. eq 11). Cluster structures are taken from the PBE+MBD optimized pbc (2 × 2), 4-layered pbc structures. Tabulated values are given in Tables S2.3 and S2.4.

Figure 3 shows the dependence of total PBE+D adsorption energies and their dispersive components on the size and shape of the cluster models. As a reference, the results obtained with periodic boundary conditions (pbc) are shown as dotted lines. Since the cluster models consider isolated adsorbates, the lateral interactions (see Table S2.3) have been removed from the pbc results. As expected, the adsorption energy stems virtually entirely from the dispersion, whereas the PBE adsorption energy is nearly zero under pbc.

The adsorption energies converge quickly with the cluster size and shape. From Pt_19_(12,7) onward, the variation is within a few kJ mol^–1^, reaching 2.6 and 2.3 kJ mol^–1^ for the largest clusters for PBE and MBD, Pt_46_ and Pt_58_ (similar for LCAO, cf. Figure S3.1).

The dependence of the dispersion correction on the cluster size for MBD on the one hand, and D3 and dDsC on the other hand, differ significantly. The former saturates rapidly, by Pt_19_, then it gets slightly less binding. This is attributed to the Coulomb screening effect, known to be strong in metals, which effectively suppresses the long-range component of dispersion. In contrast, D3 and dDsC continue to add dispersion beyond the Pt_19_ cluster, their plots being virtually parallel. This suggests that none of these two terms captures the Coulomb screening effect in the dispersion interaction, thus describing the long-range dispersion incorrectly. Even at short distances, the Coulomb screening can have a noticeable effect. For example, MBD yields stronger binding than dDsC with the smallest cluster Pt_4_, but this reverses for clusters larger than Pt_19_.

Concerning the effectiveness of the DFT model in the QM:QM scheme, the excessive unscreened dispersion in the dDsC and D3 models renders them unsuitable for metals. Indeed, in order to cancel out the unphysical long-range dispersive tails from the pbc in the hybrid RPA:DFT+D scheme, RPA calculations on very large clusters would be required. This would add unnecessary computational burdens, as well as requiring these large clusters to be readily applicable to RPA, which is not the case for our system. From the screening perspective, the CH_4_/Pt_19_ cluster is sufficient to capture most of the screened dispersion between methane and the platinum surface, so pure PBE without any dispersion correction may also be an effective low-level method in hybrid RPA:DFT for these systems.

While we have seen that the PBE and dispersion energies quickly converge with the cluster size, the situation is very different for RPA. Differences in the electronic structure of the different clusters have a strong influence on the adsorption energies obtained. Around the HOMO, there are many multiply degenerate MOs, which must be occupied together, computationally, resulting in a non-Aufbau population where lower energy singly degenerate MOs remain unoccupied. Such reference DFT calculations precludes running RPA on top of it. This may be overcome by shifting all of the virtual MOs by an energy offset, such that all the unoccupied MOs are higher in energy than all occupied MOs, i.e., an Aufbau population. Since the RPA interaction energy is very sensitive to the HOMO–LUMO gap^70,71^ (see Figure S3.2), the energy offset must be chosen with great care. Another way to obtain an Aufbau occupation is adopting a high-spin state. This works in some cases but it cannot be recommended generally, as the nature of the interaction can be affected. This is demonstrated for the triplet state of Pt_19_ or the quintet state of Pt_28_, for which hybrid RPA:DFT values deviate from those of periodic RPA (cf. Tables S4.1 and S4.2). However, among the clusters tested at least one, Pt_19_(12,7) shown in Figure 2, provides an Aufbau population in the singlet state for PBE and, hence, is readily applicable to RPA. The following hybrid QM:QM calculations adopt this Pt_19_(12,7) cluster in its singlet state.

### 5.2 Hybrid RPA:DFT(+D) – CH_4_/Pt(111)

**Figure 4:**
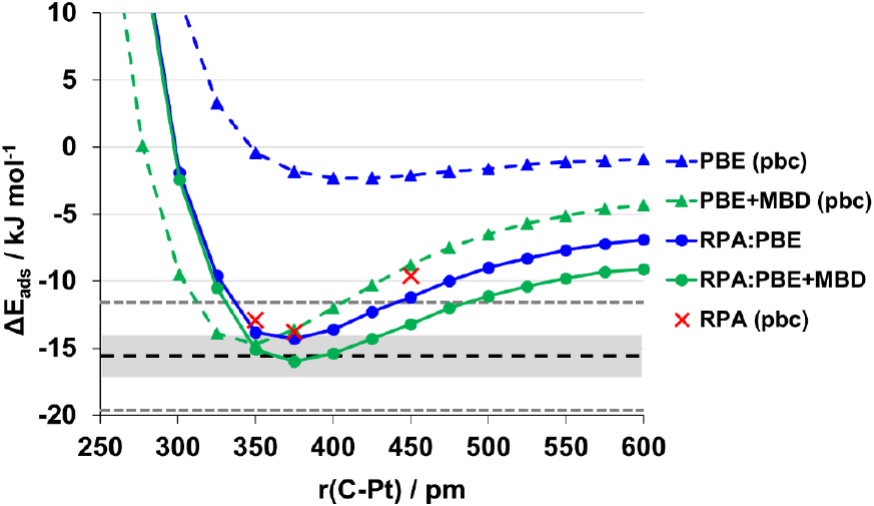
RPA:PBE and RPA:PBE+MBD adsorption energies (kJ mol^–1^) for CH_4_/Pt(111) as a function of the Pt–C distance, r(C–Pt) in pm. Red crosses are periodic RPA values; circles/full lines are hybrid RPA:DFT(+MBD) values; triangles/dashed lines are periodic values. The experiment is shown by a dashed black line with gray error bars to indicate the range of experimental error; chemical accuracy limits, ± 4 kJ mol^–1^, are shown by dashed gray lines. Points are tabulated in Table S4.3.

Figure 4 presents potential energy curves for methane adsorbed on the Pt(111) surface, using PBE and PBE+MBD directly with pbc, and as low-level methods within the hybrid RPA:DFT(+MBD) scheme. The reference RPA(pbc) values are −12.0, −13.8, and −9.6 kJ mol^–1^ for r(C–Pt) = 350, 375, and 450 pm, respectively. While PBE as expected provides only minute binding, PBE+MBD yields an adsorption energy reasonably close to the RPA(pbc) result, but noticeably underestimates the equilibrium distance. This implies that performing a DFT(+D) optimization followed by a high-level, single point calculation is of limited use for this system.

The equilibrium distances obtained with both RPA:PBE and RPA:PBE+MBD are close to the periodic RPA results (375 pm). They are also energetically quite close; however, best agreement with periodic RPA is found for hybrid RPA:PBE. Generally, RPA is known to systematically underestimate dispersive interactions,^33,72^ which may also be the case here. Interestingly, the RPA:PBE+MBD minimum is slightly deeper than the target periodic RPA reference; the MBD contributions to the hybrid then virtually match with experiment.

In Table 2, we break down the results of the hybrid RPA:PBE(+D) methods obtained at the RPA minimum into the individual components of the QM:QM scheme. As discussed above, the hybrid approach with dDsC, D3, (and D2) noticeably overestimates the adsorption energy compared to the RPA reference value. Whereas the low-level PBE+dDsC, PBE+D3, and PBE+D2 results for the adsorption energy vary over as much as 10 kJ mol^–1^, when used in the hybrid scheme they vary over 1.5 kJ mol^–1^ only. This again points to the unscreened treatment of the long-range part of the dispersion in these models, which is similar between them. As previously noted, the RPA:PBE+MBD result is very close to experiment but somewhat lower than periodic RPA.

**Table 2:** Hybrid RPA:PBE(+D) Adsorption Energies, Δ*E*_HL:LL,CPC_(pbc), for CH_4_/Pt(111) with RPA as High-Level (HL) and Different Dispersion Approaches as the Low-Level (LL) Methods^a^

Δ*E*/ kJ mol^–1^	PBE	PBE + MBD	PBE + dDsC	PBE + D3	PBE + D2
Δ*E*_LL_(pbc)	**–1.8**	**–13.6**	**–18.1**	**–22.3**	**–28.5**
Δ*E*_LL, CPC_(C)	–0.1	–10.3	–12.0	–16.8	–23.8
Δ*E*_disp_(C)	-	–10.2	–11.8	–16.6	–23.7
Δ*E*_HL,CPC_(C)	*-12.6*	*-12.6*	*-12.6*	*-12.6*	*-12.6*
ΔHL_CPC_(C)	**–12.5**	**–2.4**	**–0.7**	**4.1**	**11.2**
ΔLR(pbc,C)	*-1.7*	*-3.3*	*-6.2*	*-5.5*	*-4.7*
Δ*E*_HL:LL,CPC_(pbc)^b^	–14.3	–16.0	–18.8	–18.1	–17.3
Δ*E*_RPA_(pbc)^c^	–13.8				
Δ*E*_obs._^29,35^	–15.6				

a The structure corresponds to the RPA minimum in Figure 4 with r(C–Pt) = 375 pm.

b This can be summed from *E*_LL_(pbc) + ΔHL(C) (bold numbers) or *E*_HL_(C) + ΔLR(pbc, C) (italics), cf. eq 2.

c This work.

It is known that modeling adsorption on metallic surfaces using a cluster model alone is problematic, as the interaction energies may change dramatically from one cluster to another and deviate altogether from the periodic results. This effect is especially pronounced for chemisorption,^10−14,21^ but can also occur for molecular adsorption.^21^ In our cluster calculations, unphysical artifacts were observed in the potential curves for small r(C–Pt) (cf. Figure S4.2). Importantly, this faulty behavior cancels out in the high-level correction, ΔHL_CPC_(C), which is in line with the purely DFT-based hybrid scheme of Araujo et al.^21^ We note, however, that the correction with RPA as the high-level method, which explicitly includes the virtual manifold and is sensitive to the HOMO–LUMO gap, is more demanding to the choice of the cluster than hybrid DFT. As mentioned above, finding a cluster that does not manifest unphysical occupations or incorrect spin states may be an issue and require additional testing.

### 5.3 Hybrid RPA:DFT(+D) – C_2_H_6_/Pt(111)

**Figure 5:**
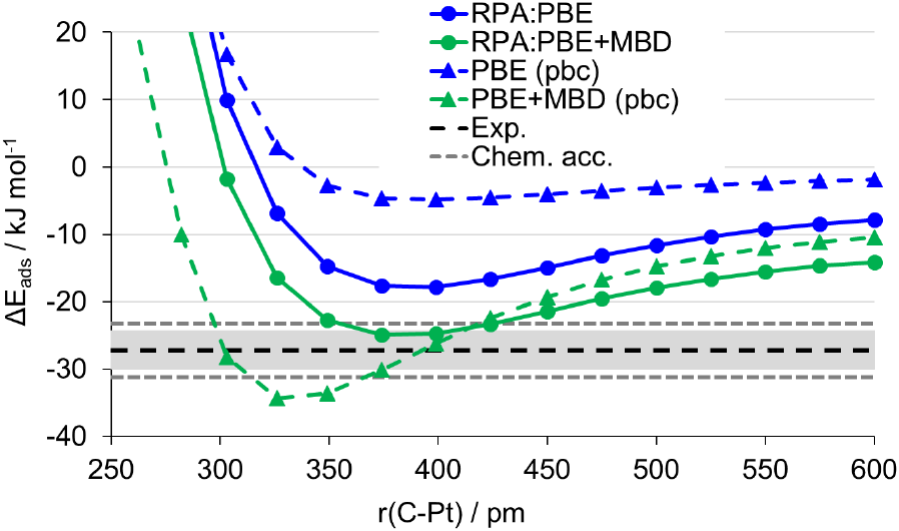
Hybrid RPA:PBE and RPA:PBE+MBD adsorption energy (kJ mol^–1^) for C_2_H_6_/Pt(111) as a function of the Pt–C distance, r(C–Pt). Circles/full lines are hybrid RPA:PBE(+MBD) values; triangles/dashed lines are periodic values. The experimentally derived reference value is shown as dashed black line with gray error bars to indicate the range of experimental uncertainty. The chemical accuracy range, ± 4 kJ mol^–1^, is shown as dashed darker gray lines. Points are tabulated in Table S4.4.

To investigate the performance of hybrid scheme for larger adsorbates, methane was substituted for ethane. The potential energy curves for the adsorption of ethane on Pt(111) are given in Figure 5. The general pattern is similar to the methane case, but the deviations become magnified.

The minimum of the potential curve for periodic PBE+MBD is further shifted to shorter distances. This stresses the importance of obtaining the optimized distance between the physisorbed molecule and the surface at a higher level of theory. Furthermore, in contrast to the methane case, ethane on platinum noticeably overestimated the interaction energy using PBE+MBD.

The hybrid approach with PBE alone gives a result that is noticeably above the experimental value, now outside the chemical accuracy error bars. RPA:PBE+MBD is again closer to experiment than RPA:PBE. It does not agree with the experiment as precisely as for methane, but is still within chemical accuracy. We do not have the periodic RPA reference for this system. Assuming a similar behavior as in the methane adsorption case, we expect the RPA:PBE result to be still closer to the periodic RPA, while the accuracy of RPA:PBE+MBD is due to error compensation. This is also in line with the usual underbinding of the van der Waals interaction by RPA. The breakdown of hybrid method energies in contributing terms is given in Table 3.

**Table 3:** Hybrid RPA:PBE and RPA:PBE+MBD Adsorption Energies, Δ*E*_HL,CPC_(pbc) for C_2_H_6_/Pt(111) at PBE+MBD Optimized Structures were Used (Pt–C Distance 374 pm)^a^

Δ*E*/kJ mol^–1^	PBE	PBE + MBD
Δ*E*_LL_(pbc)	**–4.6**	**–30.1**
Δ*E*_LL, CPC_(C)	+0.2	–18.0
Δ*E*_disp_(C)	–	–18.3
Δ*E*_HL,CPC_(C)	*–12.8*	*–12.8*
ΔHL_CPC_(C)	**–13.0**	**+5.3**
ΔLR(pbc,C)	*–4.8*	*–12.1*
Δ*E*_HL:LL,CPC_(pbc)^b^	–17.6	–24.9
Δ*E*_obsd._^c^	–27.2	

a The Pt_19_ cluster in the singlet state was used.

b This can be summed from *E*_LL_(pbc) + ΔHL(C) (bold) or *E*_HL_(C) + ΔLR(pbc, C) (italics), cf. eq 2.

c Tait et al.,^35^ see Table 1.

### 5.4 Computational Cost

The main advantage of the hybrid HL:LL approach is that it is computationally much more efficient than the high-level periodic calculation. We compare the computational times in Table 4 for CH_4_/Pt(111).

**Table 4:** CPU Times (in hr) for Periodic RPA with a 14 Å Vacuum (+ with Vacuum Extrapolation) and RPA:PBE Hybrid Approach Applied to CH_4_/Pt(111)^a^

supercell		
method	(√3 × √3)R30°	(2 × 2)
RPA(pbc)	245	876
+extrapolation	727	2618
RPA:PBE, Pt_19_-singlet	-	14

a RPA(pbc) are from Sheldon et al.^29^

We have not presented times for the hybrid calculations with the (√3 × √3)R30° supercell but we do not expect these to be very different to those for the (2 × 2) cell, as the same clusters would be used (excluding minor structural differences). This decrease of cost from 245 to ∼14 h would be a comfortable saving alone. An additional benefit is seen in that the cluster calculations effectively already include vacuum extrapolation, so this saving is even greater and in fact amounts to a decrease from 727 to 14 h. The true benefit of this hybrid approach, however, is seen for sparser coverages. Even for a supercell of similar size, the (2 × 2) cell, the cost for periodic RPA increases by hundreds of CPU hours, to 2618 h if the vacuum extrapolation is taken into account. Our hybrid approach will remain similar regardless of cell size, enabling the study of sparser cells such as (3 × 3) and (2√3 × 2√3)R30°, which are too large for the O(N^4^) algorithm to study.^33^ The low-scaling RPA algorithm O(N^3^) algorithm is capable of studying these cells but requires many CPUs and has high memory requirements, 392 CPUs and ∼3 TB RAM, respectively.^73,74^ This makes it particularly suitable for the use of huge nodes in high performance computing (HPC). Our approach, on the other hand, is more applicable to the computer clusters available in the typical group, with there being limited additional benefit from using HPC facilities for single point calculations.

We envisage that the hybrid approach for metals will be useful in applying post-HF methods to metallic systems where such methods are currently restricted. Post-HF methods have already been applied to metal clusters, where the HOMO–LUMO gap is small but nonzero.^75^

## 6 Conclusions

We have shown that a divide-and-conquer approach combining periodic models and cluster models is appropriate and cost-effective in RPA applications to alkane adsorption on the Pt(111) surface. The presented subtractive QM:QM protocol is expected to be applicable to molecular adsorption on transition metal surfaces in general. However, application of our hybrid QM:QM method to metals is far less straightforward than to nonconducting systems.^5,6^ One of the difficulties is in finding a cluster that adequately and robustly represents the adsorbate on the surface of the metal. For metals, the SCF of the underlying DFT is sensitive to size and form of the clusters and does not always converge to the physically relevant state, which in turn may affect the RPA description. Concurrently, due to very effective Coulomb screening in metals, the dispersion interactions between the surface and adsorbate are quite short-range, and so there is no need to use particularly large clusters.

The second problem is more fundamental and refers to the choice of the high-level method. RPA performs relatively well for metals but is known to systematically underestimate the van der Waals interaction. For nonmetallic systems, this problem is usually solved by evaluating a CCSD(T) correction on top of the standard high-level method, typically MP2. However, in zero- or small-gap systems, this chemically accurate model is inapplicable,^76^ and so a solution can be sought by choosing the RPA variant most appropriate for a given class of systems.^31,64,77−82^

## Supporting Information Available

Images of cluster models; tables of adsorption energies for clusters and for periodic boundary conditions; details of cluster calculations using a Gaussian basis set, DFT and RPA, including spin states; hybrid RPA:DFT(+D) tables and potential energy curves for methane and ethane (PDF)
