# Supplementary material for: Hybrid RPA:DFT Approach for Adsorption on Transition Metal Surfaces: Methane and Ethane on Platinum (111)
Source: J Chem Theory Comput. 2024 Feb 8;20(5):2219–27. doi: 10.1021/acs.jctc.3c01308 (PMC10938501; doi:10.1021/acs.jctc.3c01308)
Supplement: Supplementary file 1 — ct3c01308_si_001.pdf [file ct3c01308_si_001.pdf]

Supporting Information

Hybrid RPA:DFT Approach for Adsorption on Transition Metal Surfaces: Methane and Ethane on Platinum (111)

Christopher Sheldon,[a,b] Joachim Paier,[a,c,*] Denis Usvyat,[a] and Joachim Sauer[a,*]

[a]Institut für Chemie, Humboldt-Universität zu Berlin, Unter den Linden 6, 10099 Berlin, Germany

[b] Fritz-Haber-Institut der Max-Planck-Gesellschaft, Faradayweg 4, 14195 Berlin, Germany

[c] Lehrstuhl für Theoretische Chemie, Friedrich-Alexander-Universität Erlangen-Nürnberg, Egerlandstrasse 3, 91058 Erlangen, Germany

S1 - Cluster models

The clusters used in this study are presented in Figures S1.1, respectively. They are named in the following style: Pt_n(A,B,…), where n is the number of platinum atoms in the cluster, A is the number in the top layer of the cluster, B is the number in the second layer, and so on.

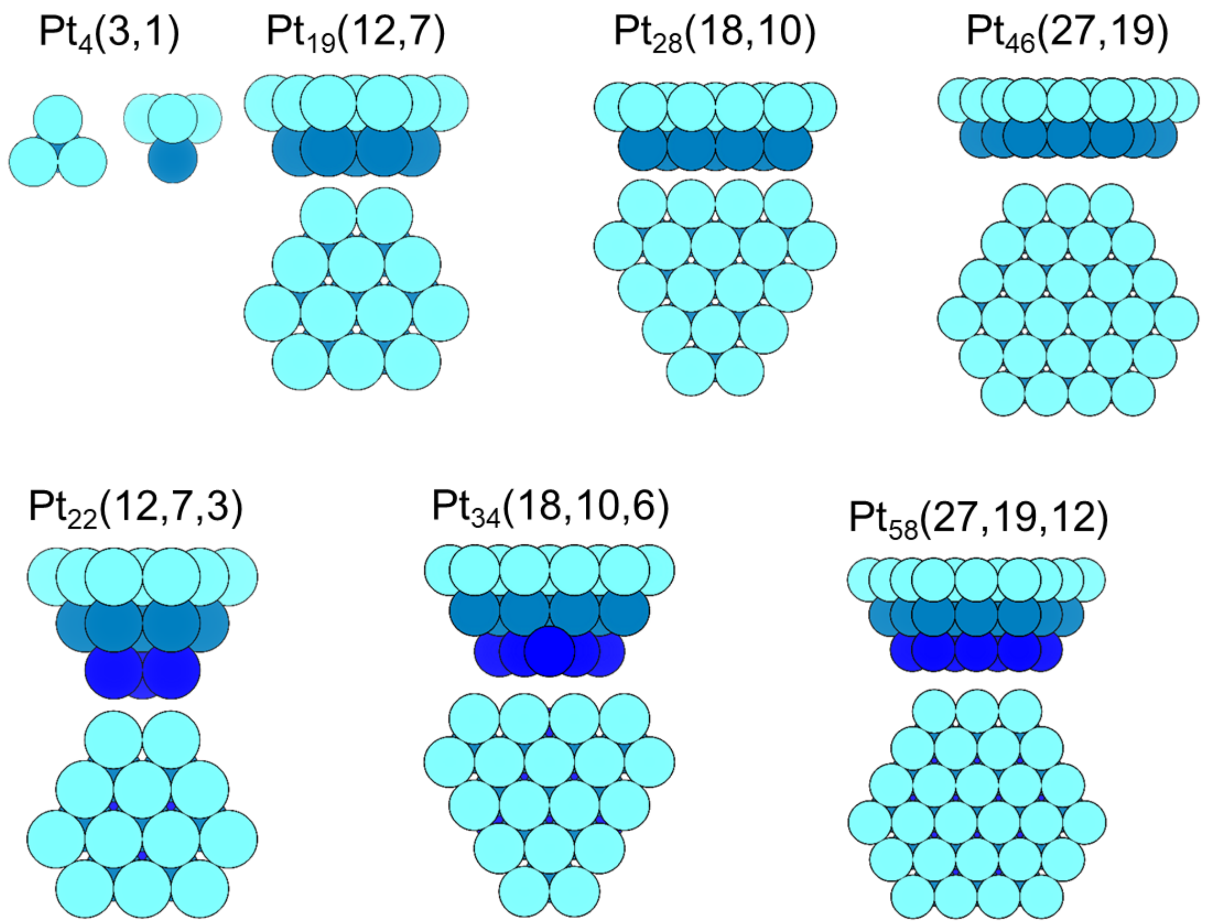

Figure S1.1. Cluster models of the surface. Light, middle, and dark blue indicate atoms in the first, second, and third layer of the cluster, respectively.[1]

S2 – Clusters under pbc

The clusters shown in Section S1 were placed into cubic cells under pbc. Table S2.1 shows the adsorption energies for the cluster in 20 Å^3 and 25 Å^3 cells.

Table S2.1. Adsorption energy ΔE_{ads} (in kJ mol^{-1}) of CH_4 on platinum clusters Pt_n(A,B,C) for PBE+MBD in 20^3 and 25^3 Å^3 cells. Where n denotes the total number of Pt atoms, A the number in the first layer, B the number in the second layer, and C the number in the third layer.

Cluster	ΔE_{ads} / kJ mol^{-1}	
	20^3	25^3
Pt_4(3,1)	-7.6	-7.6
Pt_{19}(12,7)	-11.9	-12.0
Pt_{22}(12,7,3)	-11.5	-11.5
Pt_{28}(18,10)	-12.1	-12.2
Pt_{34}(18,10,6)	-10.8	-10.9
Pt_{46}(27,19)	-9.9	-10.1
Pt_{58}(27,19,12)	-9.0	-9.1

We present the adsorption energies with and without spin-polarisation in Table S2.2 below. As the D2 and D3 dispersion corrections are post-SCF additive and not density-dependent, they are not impacted by spin polarisation besides the change in the PBE component.

Table S2.2. Spin-polarised (SP) and non-spin-polarised (non-SP) adsorption energy ΔE_{ads} (in kJ mol^{-1}) of CH_4 on platinum clusters Pt_n(A,B,C) for PBE with dispersion corrections (dDsC, and MBD).

Cluster	PBE		dDsC		MBD	
	SP	Non-SP	SP	Non-SP	SP	Non-SP
Pt_4(3,1)	0.3	0.3	-6.0	-6.1	-7.5	-7.6
Pt_{19}(12,7)	1.0	1.1	-12.8	-12.7	-12.0	-11.9
Pt_{22}(12,7,3)	1.6	1.5	-12.4	-12.5	-11.3	-11.4
Pt_{28}(18,10)	1.2	0.9	-13.7	-14.0	-12.1	-12.4
Pt_{34}(18,10,6)	2.0	1.9	-13.1	-13.2	-10.8	-10.9
Pt_{46}(27,19)	2.9	2.4	-13.2	-13.6	-9.9	-10.4
Pt_{58}(27,19,12)	2.6	2.4	-13.7	-14.0	-9.1	-9.4

Tables S2.3 and S2.4 show the adsorption energies for the clusters (Figure S1.1) with spin-polarisation under pbc.

Table S2.3. Adsorption energy, ΔE_{ads} (kJ mol^{-1}), of CH_4 on platinum clusters Pt_n(A,B,C) for PBE with dispersion corrections (D2, D3, dDsC, and MBD). Where n denotes the total number of Pt atoms, A the number in the first layer, B the number in the second layer, and C the number in the third layer. The periodic calculation is for the 3-layered (2x2) cell with lateral interactions removed, $pbc_{no\ lat}$.

Cluster		PBE+			
	PBE	D2	D3	dDsC	MBD
Pt_4(3,1)	0.3	-19.9	-10.6	-6.0	-7.5
Pt_{19}(12,7)	1.0	-31.0	-19.3	-12.8	-12.0
Pt_{22}(12,7,3)	1.6	-30.4	-18.8	-12.4	-11.3
Pt_{28}(18,10)	1.2	-32.2	-20.3	-13.7	-12.1
Pt_{34}(18,10,6)	2.0	-31.4	-19.6	-13.1	-10.8
Pt_{46}(27,19)	2.9	-31.7	-19.7	-13.2	-9.9
Pt_{58}(27,19,12)	2.6	-31.7	-20.0	-13.7	-9.1
pbc_{lat}	-0.4	-35.6	-24.9	-18.9	-14.7
ΔE_{lat}	-0.5	-1.3	-1.6	-1.6	-1.2
$pbc_{no\ lat}$	0.1	-34.3	-23.3	-17.3	-13.4

Table S2.4. Dispersion contribution to the adsorption energy, ΔE_{disp} (kJ mol^{-1}), of CH_4 on platinum clusters Pt_n(a,b,c) for PBE with different dispersion corrections. The periodic calculation is for the 3-layered (2x2) cell with lateral interactions removed, $pbc_{no\ lat}$.

Cluster	D2	D3	dDsC	MBD
Pt_4(3,1)	-20.2	-11.0	-6.4	-7.9
Pt_{19}(12,7)	-31.9	-20.3	-13.8	-13.0
Pt_{22}(12,7,3)	-32.0	-20.4	-14.0	-12.9
Pt_{28}(18,10)	-33.4	-21.5	-14.9	-13.2
Pt_{34}(18,10,6)	-33.4	-21.6	-15.1	-12.9
Pt_{46}(27,19)	-34.5	-22.6	-16.0	-12.8
Pt_{58}(27,19,12)	-34.4	-22.7	-16.4	-11.7
$pbc_{no\ lat}$	-34.4	-23.4	-17.5	-13.5

S3 – Clusters using a Gaussian Basis Set (TURBOMOLE)

S3.1 DFT

We have shown that using plane waves, i.e. a near complete basis set is appropriate for describing clusters and converges towards to pbc values. However, this corroboration does not necessarily extend smoothly to Gaussian basis sets. To investigate this, we calculated the adsorption energy for the seven different clusters using PBE with a large, Gaussian basis. The choice of state then becomes important. We chose to investigate the singlet state, as this is the physical state of bulk platinum, lacking any magnetic dipole, and the lowest energy spin state, which as commonly been used in the literature.[2] The adsorption energies are plotted against the number of Platinum atoms in the cluster in Figure S3.1. The adsorption energies were obtained using PBE/def2-QZVPP and the Basis Set Superposition Error (BSSE) was accounted for using the Counterpoise Correction (CPC).

Figure S3.1. Adsorption energy plots for CH_4 on Pt_n clusters for different sized clusters in the singlet (blue) and lowest spin (red) states using Gaussian basis sets. Circles show the total

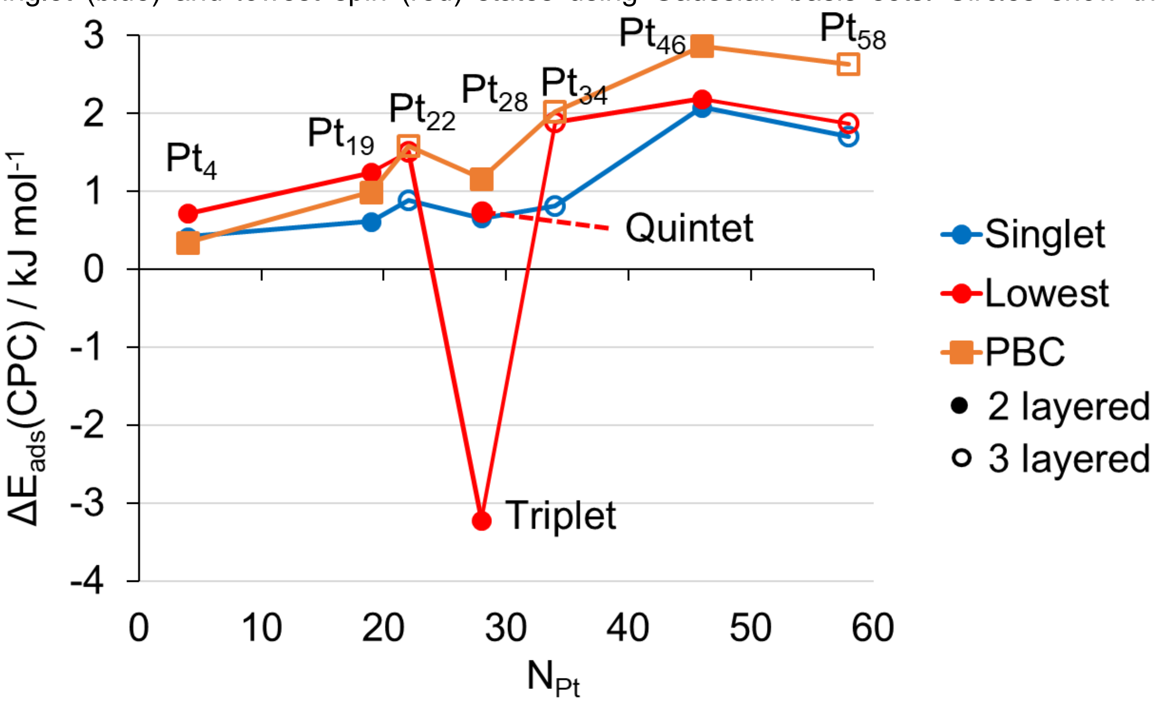

adsorption energy, while triangles show the dispersive component. Full markers are for 2-layered clusters, while empty show the markers are for 3-layered clusters. Plane wave values from Figure 3 are shown for reference (orange squares). Tabulated values given in Table S3.1. The adsorption energies were obtained using PBE/def2-QZVPP and BSSE-corrected with the Counterpoise Correction (CPC).

The difference between the adsorption energies for the singlet and lowest spin states is not significant, varying by less than 1 kJ mol^{-1} with the singlet state being consistently lower in energy. We checked the adsorption energy up to the 39-tet state for Pt_{19} and found that it did not change significantly with multiplet state, see Figure S3.3 and Table S3.2, with only a few minor deviations. We expect that this difference from the older literature, where different states gave very different adsorption energies, was due to their use of the Hartree-Fock method, which is poor for delocalised systems, such as metal clusters.[2-6] By using DFT, one can compensate for this and make the clusters suitable for describing adsorption on surfaces.[7,8]

One important exception is for the $Pt_{18,10}$ cluster, where the lowest energy spin state (the triplet in this case) is significantly lower in energy. Upon investigation, it became clear that this is due to close-lying triplet states that are difficult to distinguish. Instead, we show the quintet state for this is far closer to the singlet state and does not significantly deviate, so we will use this in subsequent calculations on this cluster.

Once again, we see little difference between the 2- and 3-layered clusters, generally less than 0.5 kJ mol^{-1}, confirming our suspicion that there is no real benefit in the use of additional layers, so we will not consider additional layers further. The general trend of adsorption energies with respect to cluster size is similar for both the singlet and lowest spin states, showing similar curves. This is matched by the trend for those adsorption energies obtained from the plane wave basis set, which indicates that they describe the electronic structure similarly. This corroborates that either a plane wave or atom-centred, Gaussian is suitable for the description of these platinum clusters using DFT(+D). This makes it appropriate to use as the low-level method in a hybrid scheme.

3.2.2 RPA

Few post-HF methods are suitable for studying metals, due to their zero band-gap. One such method is the Random Phase Approximation (RPA). This has successfully been applied to several systems with adsorption on surfaces under periodic settings.[9-11] Alternatively, clusters may be used.[8] As these are only mimics for the surface, they do not have an exactly zero band-gap, so other methods may also be suitable, if they were also computationally feasible. We have applied RPA to the metal clusters investigated previously and encountered additional problems. Although the band-gap is not as severe an issue as might have been expected, there is nonetheless great difficulty in finding a suitable spin state for calculating. Above we tested the singlet and the lowest energy spin states and found that they worked well for DFT. We then took these PBE orbitals and

used them for RPA. We found that there is a strong dependence on the HOMO-LUMO gap,[12,13] the non-periodic analogue of the band-gap. To test this, we took a cluster that performed well, the singlet state of the Pt_{19} cluster and set the HOMO-LUMO gap to a set value by shifting all the energy of the virtual orbitals by the same amount, then performed RPA calculations. N.B. the PBE orbitals remained otherwise unchanged. We show the adsorption energy against the HOMO-LUMO gap for CH_4/Pt_{19} using RPA in Figure S3.2.

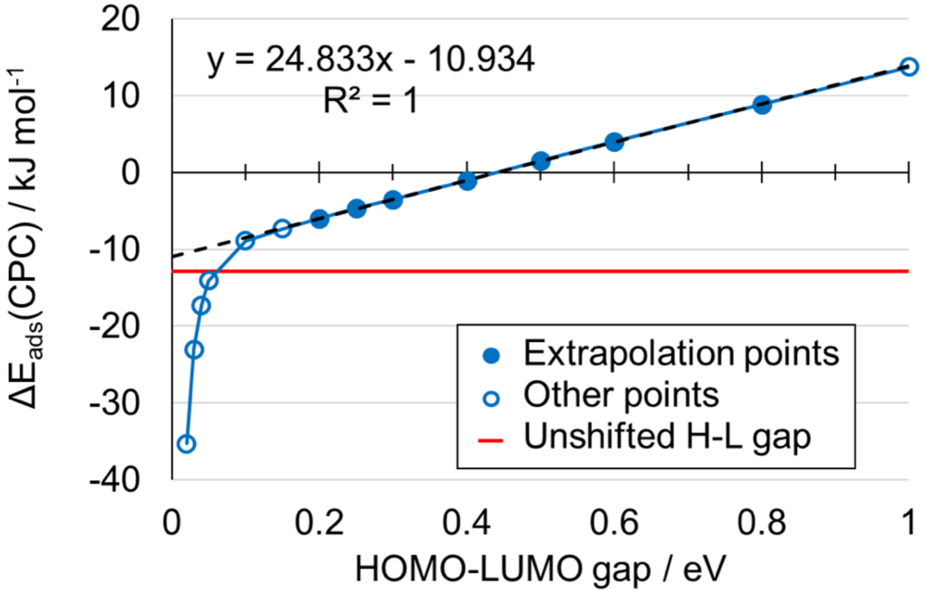

Figure 3.2. RPA adsorption energy (in kJ mol^{-1}) against the HOMO-LUMO gap (in eV) for singlet CH_4/Pt_{19}. Points are tabulated in Table S3.3. The red line is the adsorption energy without any shifting of the HOMO-LUMO gap.

It is clear from Figure 3.2 that the adsorption energy is linear with respect to the HOMO-LUMO gap beyond 0.2 eV, leading us to wonder whether this could be extrapolated to a “zero-gap” value to better mimic the metal surface. This did not match the unshifted HOMO-LUMO gap value (-12.9 kJ mol^{-1}), however, instead underestimating it by 2 kJ mol^{-1}. Additionally, it is clear that this implementation of RPA (as an approximation of ring CCD)[14] is not immune from the zero-gap issue, unlike periodic RPA. Instead, the adsorption energy becomes increasingly strong as the HOMO-LUMO gap tends towards zero, resulting in unphysically strong binding. However, so long as a small, non-zero HOMO-LUMO gap is found for the cluster, good RPA adsorption energies may still be performed.

We tested the HOMO-LUMO gaps for all our clusters and found that, with the exception of the Pt_{19} cluster, the singlet state resulted in a negative HOMO-LUMO gap, i.e. a non-Aufbau

population, and making it inappropriate for further use. This can be amended by forcing a final diagonalisation of the Fock matrix. However, this introduces an artificially large HOMO-LUMO gap, rendering the RPA adsorption energies meaningless (cf. dependency of ΔE_{ads} in Figure S3.2 and Table S3.4). This is due to doubly-degenerate orbitals being populated preferentially before singly degenerate, resulting in a hole in the orbital population. However, the singlet state of the Pt_{19} cluster is suitable, due to the HOMO being singly, rather than doubly, degenerate. Additionally, triplet and other multiplet states are suitable in every case. However, due to RPA calculations becoming computationally intractable for the larger clusters, we limit ourselves to the Pt_{19} and Pt_{28} clusters. The adsorption energies for different clusters using a Gaussian basis (Figure S3.1) are presented in Table S3.1.

Table S3.1. Adsorption energy ΔE_{ads} (in kJ mol^{-1}) for CH_4 on platinum clusters Pt_n(A,B,C) in the singlet states and the lowest energy spin states using PBE/def2-QZVPP, and the plane wave (PW) value. Comparison is made against the Singlet state, so the non-spin-polarised PW values are given. The lowest energy spin state multiplicity $(2S+1)_{low}$, Counterpoise-corrected (CPC) adsorption energies, and the Basis Set Superposition Error (BSSE) are given. Additionally, the values for the quintet state of Pt_{28} are shown.

Cluster	$(2S+1)_{low}$	ΔE_{ads} / kJ mol^{-1}				
		Singlet		Lowest		PW
		CPC	BSSE	CPC	BSSE	
Pt_4(3,1)	3	0.4	-0.3	0.7	-0.3	0.3
Pt_{19}(12,7)	7	0.6	-0.2	1.2	-0.2	1.1
Pt_{22}(12,7,3)	13	0.9	-0.2	1.5	-0.2	1.5
Pt_{28}(18,10)	3	0.7	-0.2	-3.2	-0.2	0.9
(quintet)	(5)	-	-	0.7	-0.2	-
Pt_{34}(18,10,6)	13	0.8	-0.3	1.9	-0.3	1.9
Pt_{46}(27,19)	19	2.1	-0.3	2.2	-0.3	2.4
Pt_{58}(27,19,12)	19	1.7	-0.3	1.9	-0.3	2.4

The adsorption energies for different spin states of Pt_{19} are given in Table S3.2 and shown in Figure S3.3.

Table S3.2. Adsorption energy ΔE_{ads} (in kJ mol^{-1}) for CH_4 on Pt_{19} clusters in multiplet states using PBE/def2-QZVPP. The Counterpoise-corrected (CPC) adsorption energies, and the Basis Set Superposition Error (BSSE) are given.

2S+1	ΔE_{ads}(CPC) / kJ mol^{-1}
1	0.6
3	0.8
5	2.5
7	1.2
9	0.8
11	0.8
13	0.4
15	1.0
17	1.0
19	1.1
21	1.1
23	1.2
25	1.2
27	1.1
29	-0.2
31	0.5
33	0.0
35	4.6
37	3.8
39	5.0

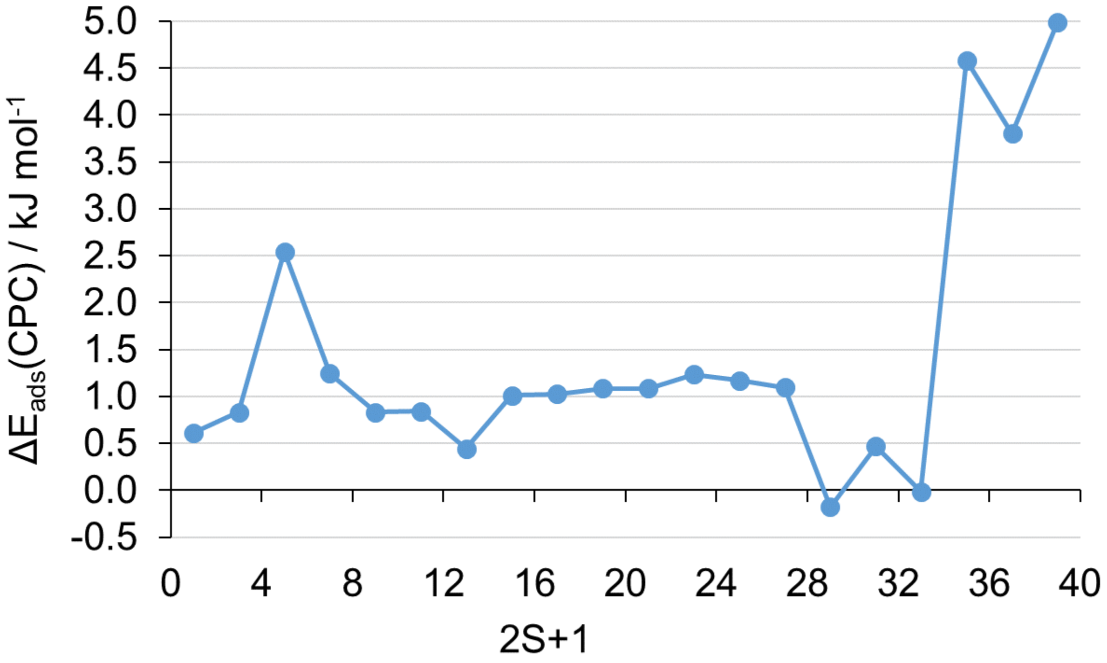

Figure S3.3. Adsorption energy ΔE_{ads} (in kJ mol^{-1}) for CH_4/Pt_{19} against multiplicity using PBE/def2-QZVPP.

The RPA adsorption energies for CH_4/Pt_{19} are presented in Table S3.3. For these calculations, the HOMO-LUMO gap for Pt_{19}, CH_4/Pt_{19}, $Pt_{19}//CH_4/Pt_{19}$, and $Pt_{19}(CH_4)//CH_4/Pt_{19}$ were set to stated value, shifting all the virtual orbitals by the same amount as the LUMO, and then the RPA calculation was performed.

Table S3.3. Adsorption energy ΔE_{ads} (in kJ mol^{-1}) for CH_4/Pt_{19} clusters with set HOMO-LUMO gaps using RPA/def2-QZVPP. BSSE has been done according to CPC.

HOMO-LUMO gap / eV	ΔE_{ads}(CPC) / kJ mol^{-1}
0.01	-274.9
0.02	-35.3
0.03	-23.0
0.04	-17.3
0.05	-14.1
0.1	-8.9
0.15	-7.3
0.2	-6.0
0.25	-4.7
0.3	-3.5
0.4	-1.0
0.5	1.5
0.6	4.0
0.8	8.9
1	13.7
1.25	19.6
1.5	25.5
2	36.9
3	58.7

The HOMO-LUMO gap for cluster calculations, with number of Pt atoms given, is shown in Tables S3.4 for the singlet states.

Table S3.4. HOMO-LUMO gaps (in eV) for PBE/def2-QZVPP for n-atom clusters in the singlet state.

N_{Pt}	HOMO-LUMO Gap / eV			
	Pt_n	$Pt_n//CH_4/Pt_n$	$Pt_n(CH_4)//CH_4/Pt_n$	CH_4/Pt_n
4	10.37	0.07	8.06	0.07
19	0.05	0.05	0.05	0.05
28	1.14	0.35	1.65	0.01
46	3.59	1.27	5.01	6.32
22	0.24	2.65	0.41	0.06
34	0.13	0.02	0.00	0.00
58	1.45	8.49	0.09	0.03

The HOMO-LUMO gap for cluster calculations against the number of Pt atoms is presented in Figure S3.4 for singlet and triplet states.

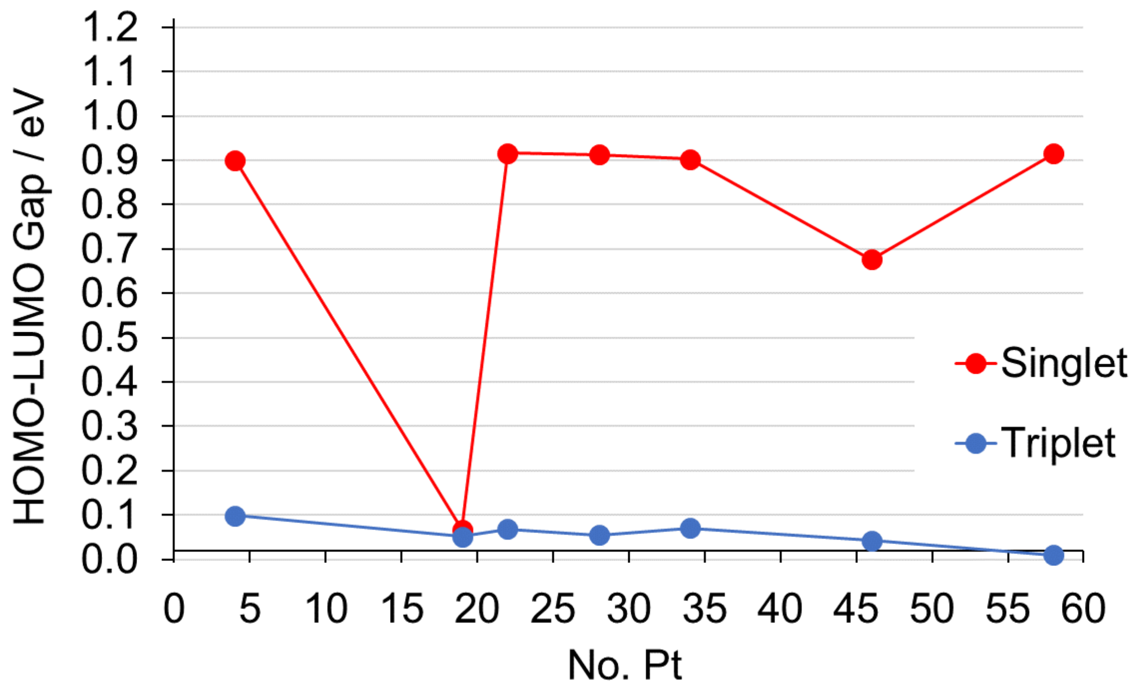

Figure S3.4. HOMO-LUMO gap (in eV) for Pt clusters against number of Pt atoms using PBE/def2-QZVPP for singlet (red) and triplet (blue) states.

S4 – Hybrid RPA:DFT

Methane. The hybrid adsorption energies for Pt_{19} in the triplet state are shown and broken down in Table S4.1.

Table S4.1. Hybrid RPA:PBE(+D) adsorption energy $\Delta E_{HL:LL,CPC}$(pbc) for methane on Pt(111) at the PBE+MBD optimised structure (note a different r(C-Pt) compared to Table 2 of the main text). The Pt_{19} cluster in the triplet state was used.

ΔE / kJ mol^{-1}	PBE	PBE+MBD	PBE+dDsC	PBE+D3	PBE+D2
ΔE_{LL}(pbc)	0.1	-14.7	-18.9	-24.9	-35.6
$\Delta E_{LL, CPC}$(C)	0.3	0.3	0.3	0.3	0.3
ΔE_{disp}(C)	0.0	-13.0	-13.8	-20.3	-31.9
$\Delta E_{LL, CPC}$(C) + ΔE_{disp}(C, VASP)	0.3	-12.7	-13.5	-20.0	-31.7
$\Delta E_{HL,CPC}$(C)	-8.5	-8.5	-8.5	-8.5	-8.5
ΔHL_{CPC}(C)	-8.8	4.2	5.0	11.5	23.2
ΔLR(pbc,C)	-0.2	-2.0	-5.4	-4.9	-4.0
$\Delta E_{HL:LL,CPC}$(pbc)	-8.6	-10.4	-13.9	-13.4	-12.4
ΔE_{RPA}(pbc)	-12.8[15]				
$\Delta E_{obs.}$	-15.6[15]				

The hybrid adsorption energies for Pt_{28} in the quintet state are shown and broken down in Table S4.2.

Table S4.2. Hybrid RPA:PBE(+D) adsorption energy $\Delta E_{HL:LL,CPC}$(pbc) for methane on Pt(111) at the PBE+MBD optimised structure (note a different r(C-Pt) compared to Table 2 of the main text). The Pt_{28} cluster in the quintet state was used.

ΔE / kJ mol^{-1}	PBE	PBE+MBD	PBE+dDsC	PBE+D3	PBE+D2
ΔE_{LL}(pbc)	0.1	-14.7	-18.9	-24.9	-35.6
$\Delta E_{LL, CPC}$(C)	0.7	0.7	0.7	0.7	0.7
ΔE_{disp}(C)	0.0	-13.2	-14.9	-21.5	-33.4
$\Delta E_{LL, CPC}$(C) + ΔE_{disp}(C, VASP)	0.7	-12.5	-14.2	-20.8	-32.6
$\Delta E_{HL,CPC}$(C)	-8.1	-8.1	-8.1	-8.1	-8.1
ΔHL_{CPC}(C)	-8.8	4.4	6.1	12.7	24.6
ΔLR(pbc,C)	-0.6	-2.2	-4.7	-4.2	-3.0
$\Delta E_{HL:LL,CPC}$(pbc)	-8.7	-10.2	-12.8	-12.2	-11.0
ΔE_{RPA}(pbc)	-12.8[15]				
$\Delta E_{obs.}$	-15.6[15]				

The carbon-platinum distance r(C-Pt) was then varied and then reoptimized on the PBE+MBD level with the C atom frozen in place. The adsorption energy using the hybrid structure for these methods is shown in Table S4.3 below.

Table S4.3. Hybrid RPA:PBE(+D) adsorption energies $\Delta E_{HL:LL,CPC}$(pbc) for methane on Pt(111) as function of the Pt-C distance, r(C-Pt) in pm, at the PBE+MBD optimised structure. (C) refers to the Pt_{19} cluster in the singlet state.

r(C-Pt)	PBE	PBE+ MBD	PBE+d DsC	PBE+ D3	PBE+ D2	PBE (C)	PBE+ MBD (C)	RPA (C)	RPA (pbc)
253	44.5	42.9	40.6	35.4	33.2	2.7	-25.2	-2.6	-
277	15.6	14.7	11.3	8.3	7.3	-1.0	-24.8	-10.2	-
301	-1.9	-2.4	-6.5	-7.8	-8.0	-0.5	-20.5	-13.5	-
325	-9.6	-10.5	-14.3	-14.5	-14.2	-0.1	-16.5	-13.1	-
350	-13.8	-15.1	-18.5	-18.1	-17.4	0.6	-12.4	-12.9	-12.9
375	-14.3	-16.0	-18.8	-18.1	-17.3	-0.1	-10.3	-12.6	-13.8
400	-13.6	-15.4	-17.7	-17.0	-16.1	0.1	-7.8	-11.2	-
425	-12.3	-14.3	-14.8	-15.5	-14.5	-0.3	-6.3	-10.4	-
450	-11.2	-13.2	-14.4	-14.1	-13.0	0.1	-4.6	-9.0	-9.6
475	-10.0	-12.0	-12.9	-12.7	-11.5	0.5	-3.1	-7.6	-
500	-9.0	-11.1	-11.7	-11.6	-10.4	0.5	-2.4	-7.0	-
525	-8.3	-10.4	-10.8	-10.8	-9.6	0.5	-1.8	-6.5	-
550	-7.7	-9.8	-10.1	-10.1	-8.9	0.4	-1.4	-6.1	-
575	-7.2	-9.3	-9.5	-9.5	-8.3	0.4	-1.2	-5.8	-
600	-6.9	-9.1	-9.2	-9.3	-8.0	0.4	-0.9	-5.6	-

The potential energy curves for CH_4/Pt(111) using PBE+D2, PBE+D3, and PBE+dDsC are plotted in Figure S4.1.

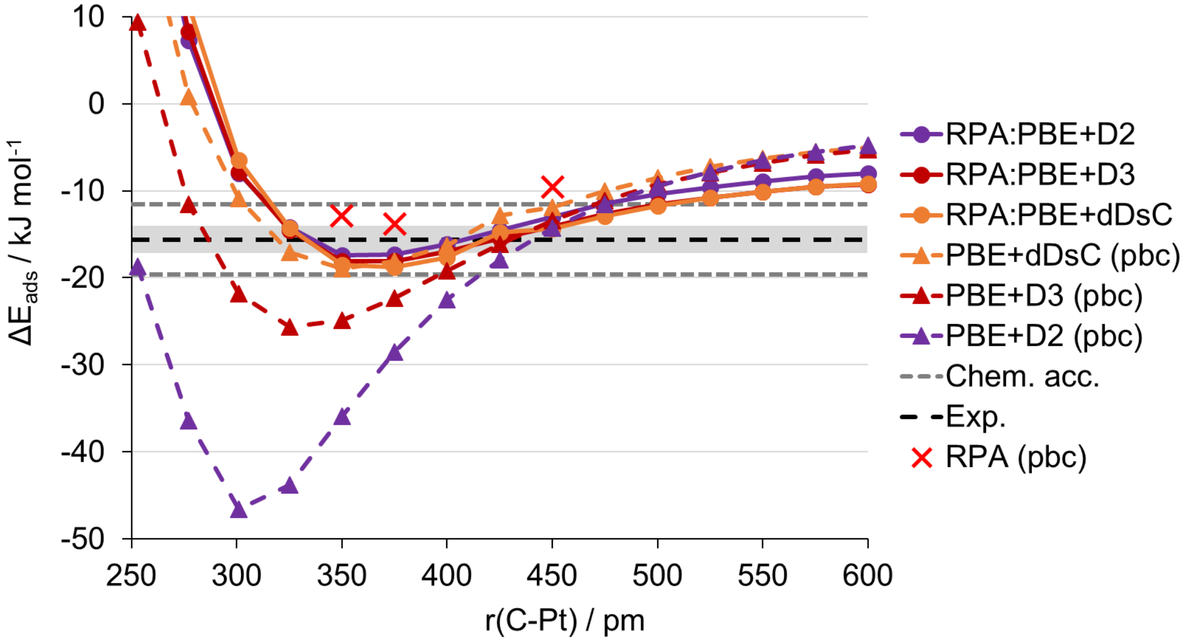

Figure S4.1. RPA:PBE+D (D2, D3, dDsC) adsorption energy (in kJ mol^{-1}) against Pt-C distance r(C-Pt) in pm for CH_4/Pt(111). Red crosses are periodic RPA values; circles/ full lines are hybrid values; triangles/ dashed lines are periodic values. The experiment is shown by a dashed black line with grey error bars to indicate the range of experimental error; chemical accuracy, ±4 kJ mol^{-1} is shown by dashed darker grey lines. Points are tabulated in Table S4.3.

The potential energy curve for CH_4/Pt(111) using PBE:RPA and PBE+MBD:RPA are plotted in Figure S4.2, alongside the adsorption energies for CH_4/Pt_{19} using PBE, PBE+MBD, and RPA.

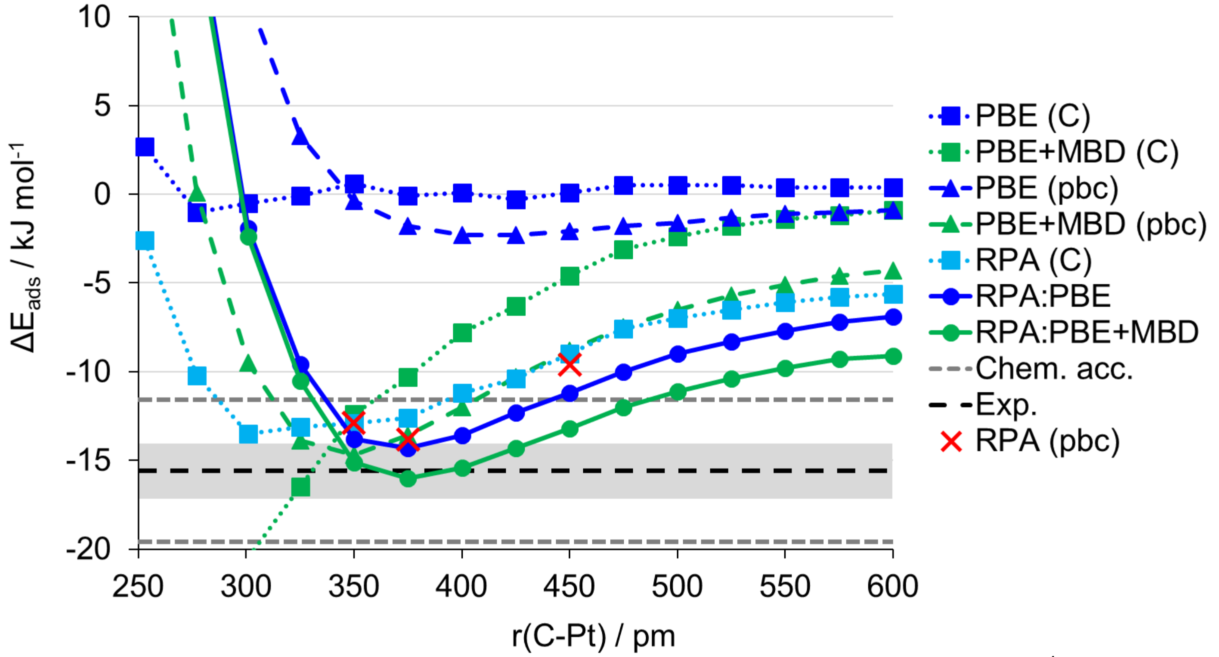

Figure S4.2. Hybrid RPA:PBE and RPA:PBE+MBD adsorption energies (kJ mol^{-1}) as function of the Pt-C distance, r(C-Pt), for CH_4/Pt(111), along with breakdown of cluster terms. Red crosses are periodic RPA values; circles/ full lines are hybrid values; triangles/ dashed lines are periodic values; squares are cluster components (C). The experiment is shown by a dashed black line with grey error bars to indicate the range of experimental error; chemical accuracy, ±4 kJ mol^{-1} is shown by dashed darker grey lines. Points are tabulated in Table S4.3.

Ethane

The carbon-platinum distance r(C-Pt) was then varied and then reoptimized on the PBE+MBD level with the C atom frozen in place. The adsorption energy using the hybrid structure for these methods is shown in Table S4.4 below.

Table S4.4. Hybrid RPA:PBE(+D) adsorption energies $\Delta E_{HL:LL,CPC}$(pbc) for C_2H_6/Pt(111) as function of the Pt-C distance, r(C-Pt) in pm, at the PBE+MBD optimised structure. (C) refers to the Pt_{19} cluster in the singlet state.

r(C-Pt) pm	PBE	PBE+MBD	PBE+ dDsC	PBE+ D3	PBE+ D2	PBE (C)	PBE+ MBD (C)	RPA (C)
260	87.9	72.7	73.3	57.9	35.9	15.1	-29.6	20.4
282	39.3	25.7	22.3	14.7	-0.8	-0.7	-39.7	-4.0
303	9.9	-1.7	-5.4	-9.4	-18.3	-5.1	-38.4	-11.9
326	-6.8	-16.4	-22.3	-22.1	-26.4	-3.2	-31.0	-13.1
349	-14.7	-22.7	-31.3	-27.6	-29.3	-0.9	-23.6	-12.8
374	-17.6	-24.9	-33.8	-29.3	-30.1	0.2	-18.0	-12.8
399	-17.8	-24.7	-33.4	-28.7	-29.2	0.5	-14.0	-12.5
424	-16.6	-23.3	-30.3	-27.1	-27.6	0.5	-10.8	-11.6
450	-14.9	-21.4	-26.5	-25.0	-25.6	0.3	-8.4	-10.5
475	-13.1	-19.5	-24.0	-23.0	-23.8	0.1	-6.7	-9.5
500	-11.6	-17.9	-21.9	-21.3	-22.2	0.0	-5.4	-8.5
525	-10.3	-16.6	-22.4	-19.9	-20.9	0.0	-4.3	-7.7
550	-9.2	-15.5	-20.0	-18.7	-19.8	-0.1	-3.6	-7.1
575	-8.4	-14.6	-19.0	-17.8	-19.0	-0.1	-2.9	-6.4
600	-7.8	-14.1	-18.3	-17.2	-18.4	-0.1	-2.4	-6.0

The potential energy curve for C_2H_6/Pt(111) using PBE:RPA and PBE+MBD:RPA are plotted in Figure S4.3, alongside the adsorption energies for C_2H_6/Pt_{19} using PBE, PBE+MBD, and RPA.

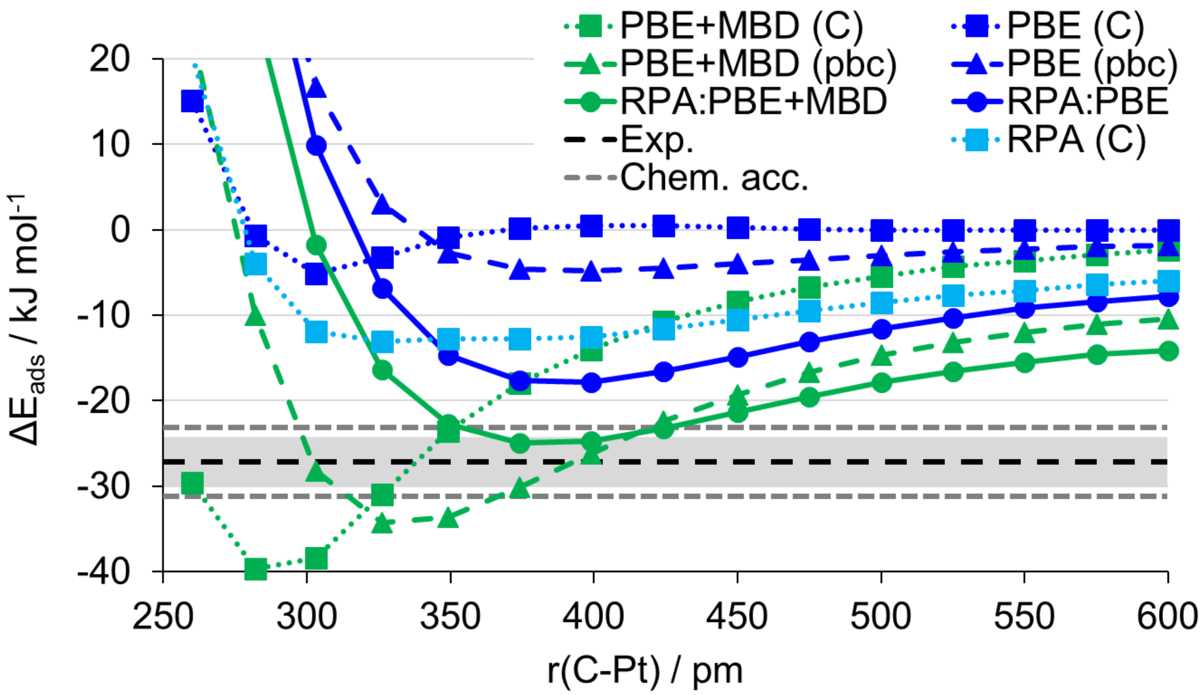

Figure S4.3. RPA:PBE and RPA:PBE+MBD adsorption energy (in kJ mol^{-1}) against Pt-C distance r(C-Pt) in pm for C_2H_6/Pt(111), along with breakdown of cluster terms. Circles/ full lines are hybrid values; triangles/ dashed lines are periodic values; squares are cluster components (c). The experiment is shown by a dashed black line with grey error bars to indicate the range of experimental error; chemical accuracy, ±4 kJ mol^{-1} is shown by dashed darker grey lines. Points are tabulated in Table S4.4.

A comparison between the adsorption energies for ethane on Pt(111) using different clusters and spin states is presented in Table S4.5.

Table S4.5. Breakdown of the adsorption energy $\Delta E_{HL:LL,CPC}$(pbc) of C_2H_6/Pt(111) for RPA:PBE(+MBD). The Pt_{19} (singlet) and Pt_{28} (quintet) clusters were used.

ΔE / kJ mol^{-1}	r(C-Pt) / pm			
	375		450	
	Pt_{19}	Pt_{28}	Pt_{19}	Pt_{28}
$\Delta E_{PBE,\ CPC}$(C)	0.2	2.6	0.3	0.6
$\Delta E_{PBE+MBD,\ CPC}$(C)	-18.0	-16.2	-8.4	-8.5
$\Delta E_{RPA,\ CPC}$(C)	-12.8	-10.8	-10.5	-9.2
$\Delta E_{RPA:PBE,\ CPC}$(pbc)	-17.6	-18.0	-14.9	-13.8
$\Delta E_{RPA:PBE+MBD,\ CPC}$(pbc)	-24.9	-24.7	-21.4	-20.0

The number of unoccupied bands used for the RPA calculations has, thus far, not been considered. We present these below in Table S4.6 for both VASP and TURBOMMOLE calculations. N.B. VASP, i.e. plane wave calculations, require far more virtual orbitals to converge the energy.

Table S4.6. Number of frozen and active occupied orbitals N_{occ}, virtual orbital N_{virt}, and total orbitals N_{total} using VASP (a plane wave PW code) and TURBOMOLE (T'MOLE, a Linear Combination of Atomic Orbitals LCAO code) for CH_4 and C_2H_6 on the Pt(111) surface; calculations in VASP used a (2x2) cell, and TURBOMOLE used a Pt_{19} or Pt_{28} cluster in the Singlet (S) or Quintet (Qu) states, respectively.

Functions (Software)	System	R_{vac} (Å)	N_{occ} *frozen*[a]	N_{occ} *active*	N_{virt}	N_{total}
PW (VASP)	Pt(111)	10.3	408	60	10084	10144
		11.3	408	60	10756	10816
		12.3	408	60	11460	11520
		13.3	408	60	12132	12192
		14.3	408	60	12868	12928
	CH_4/Pt(111)	10.3	409	64	10080	10144
		11.3	409	64	10752	10816
		12.3	409	64	11456	11520
		13.3	409	64	12128	12192
		14.3	409	64	12864	12928
LCAO (T'MOLE)	Pt_{19} (S)		76	95	1501	1672
	Pt_{28} (Qu)		224	280	4424	4928
	CH_4/Pt_{19} (S)		77	99	1673	1849
	C_2H_6/Pt_{19} (S)		78	102	1786	1966
	C_2H_6/Pt_{28} (Qu)		228	294	4994	5516

[a] frozen orbitals for PW calculations are considered to be those described by a pseudopotential, hence why $N_{total} = N_{occ, active} + N_{virt}$ in these cases.

S5 References

1. Momma, K.; Izumi, F., VESTA 3 for three-dimensional visualization of crystal, volumetric and morphology data. *J. Appl. Crystallogr.* **2011,** *44*, 1272-1276, https://doi.org/10.1107/S0021889811038970
2. Panas, I.; Schüle, J.; Siegbahn, P.; Wahlgren, U., On the cluster convergence of chemisorption energies. *Chem. Phys. Lett.* **1988,** *149*, 265-272, https://doi.org/10.1016/0009-2614(88)85024-3.
3. Nygren, M. A.; Siegbahn, P. E. M., Theoretical study of chemisorption of carbon monoxide on copper clusters. *J. Phys. Chem.* **1992,** *96*, 7579-7584, https://doi.org/10.1021/j100198a018.
4. Pettersson, L. G. M.; Faxen, T., Massively parallel direct SCF calculations on large metal clusters: Ni5-Ni481. *Theor. Chim. Acta* **1993,** *85*, 345-361, https://doi.org/10.1007/BF01113428.
5. Witko, M.; Hermann, K., Site-dependent binding of methoxy on Cu(111): Cluster model studies. *J. Chem. Phys.* **1994,** *101*, 10173-10180, https://doi.org/10.1063/1.468006.
6. Triguero, L.; Wahlgren, U.; Boussard, P.; Siegbahn, P., Calculations of hydrogen chemisorption energies on optimized copper clusters. *Chem. Phys. Lett.* **1995,** *237*, 550-559, https://doi.org/10.1016/0009-2614(95)00353-6.
7. Gil, A.; Clotet, A.; Ricart, J. M.; Kresse, G.; Garcı; x; a-Hernández, M.; Rösch, N.; Sautet, P., Site preference of CO chemisorbed on Pt(111) from density functional calculations. *Surf. Sci.* **2003,** *530*, 71-87, https://doi.org/10.1016/S0039-6028(03)00307-8.
8. Ren, X.; Rinke, P.; Scheffler, M., Exploring the random phase approximation: Application to CO adsorbed on Cu(111). *Phys. Rev. B* **2009,** *80*, 045402, https://doi.org/10.1103/PhysRevB.80.045402.
9. Schmidt, P. S.; Thygesen, K. S., Benchmark Database of Transition Metal Surface and Adsorption Energies from Many-Body Perturbation Theory. *J. Phys. Chem. C* **2018,** *122*, 4381-4390, https://doi.org/10.1021/acs.jpcc.7b12258.
10. Schimka, L.; Harl, J.; Stroppa, A.; Grüneis, A.; Marsman, M.; Mittendorfer, F.; Kresse, G., Accurate surface and adsorption energies from many-body perturbation theory. *Nat. Mater.* **2010,** *9*, 741, https://doi.org/10.1038/nmat2806.
11. Garrido Torres, J. A.; Ramberger, B.; Früchtl, H. A.; Schaub, R.; Kresse, G., Adsorption energies of benzene on close packed transition metal surfaces using the random phase approximation. *Phys. Rev. Mater.* **2017,** *1*, 060803, https://doi.org/10.1103/PhysRevMaterials.1.060803.

12. Heßelmann, A.; Görling, A., Correct Description of the Bond Dissociation Limit without Breaking Spin Symmetry by a Random-Phase-Approximation Correlation Functional. *Phys. Rev. Lett.* **2011,** *106*, 093001, https://doi.org/10.1103/PhysRevLett.106.093001.

13. Caruso, F.; Rohr, D. R.; Hellgren, M.; Ren, X.; Rinke, P.; Rubio, A.; Scheffler, M., Bond Breaking and Bond Formation: How Electron Correlation is Captured in Many-Body Perturbation Theory and Density-Functional Theory. *Phys. Rev. Lett.* **2013,** *110*, 146403, https://doi.org/10.1103/PhysRevLett.110.146403.

14. Eshuis, H.; Yarkony, J.; Furche, F., Fast computation of molecular random phase approximation correlation energies using resolution of the identity and imaginary frequency integration. *J. Chem. Phys.* **2010,** *132*, 234114, https://doi.org/10.1063/1.3442749.

15. Sheldon, C.; Paier, J.; Sauer, J., Adsorption of CH4 on the Pt(111) surface: Random phase approximation compared to density functional theory. *J. Chem. Phys.* **2021,** *155*, 174702, https://doi.org/10.1063/5.0071995.
